# Multicenter Retrospective Study of *Spiroplasma ixodetis* Infantile Cataract in 8 Countries in Europe

**DOI:** 10.3201/eid3106.240954

**Published:** 2025-06

**Authors:** Luc Van Os, Nathalie Cassoux, Symira Cholidis, Pascal Dureau, Navid Farassat, Fabienne Catherine Fierz, Ebba Ghyczy, Elena-Cristina Nitulescu, Eva Stifter, Marie-José Tassignon, Anne Le Flèche-Matéos, Birgit Lorenz, for the Spiroplasma Infantile Cataract Group

**Affiliations:** University Hospital Antwerp, Edegem, Belgium (L. Van Os, M. Tassignon); University of Antwerp, Antwerp, Belgium (L. Van Os, M.-J. Tassignon); Institut Curie, Paris, France (N. Cassoux); Université Paris Descartes, Paris (N. Cassoux); Oslo University Hospital, Oslo, Norway (S. Cholidis); Rothschild Foundation Hospital, Paris (P. Dureau); Medical Center–University of Freiburg, Freiburg, Germany (N. Farassat); University Hospital Zurich, Zurich, Switzerland (F.C. Fierz); Cantonal Hospital Winterthur, Winterthur, Switzerland (F.C. Fierz); Amsterdam University Medical Centers, Amsterdam, the Netherlands (E. Ghyczy); Emergency Children’s Hospital Marie Sklodowska Curie, Bucharest, Romania (E.-C. Nitulescu); Medical University of Vienna, Vienna, Austria (E. Stifter); Institut Pasteur, Paris (A. Le Fleche-Matéos); Justus Liebig University Giessen, Giessen, Germany (B. Lorenz)

**Keywords:** Cataract, uveitis, *Spiroplasma ixodetis*, infants, bacteria, TORCHES, Europe, Belgium, France, Norway, Germany, Switzerland, Austria, the Netherlands, Romania, Luxembourg

## Abstract

*Spiroplasma ixodetis* has been reported to cause the rare combination of cataract and uveitis in infants. Through a retrospective analysis of available literature and additional unpublished cases, we identified 28 eyes from 18 infants from 8 countries in Europe with cataracts and intraocular inflammation. The cataracts were bilateral in 55.6%, unilateral in 44.4%, and progressive in 46.4% of patients. Granulomatous anterior uveitis was found in all infants. Presence of *S. ixodetis* was supported by PCR (positive in 89.3% of eyes tested), transmission electron microscopy (positive in 90% of eyes tested), or culture of aspirated lens material (positive in 87.5% of eyes tested). Treatment with macrolide antimicrobial drugs, corticosteroids, and lensectomy appeared to be effective. Two patients had a recurrence of the uveitis after lens extraction and needed prolonged treatment. To increase awareness of *S. ixodetis*, we suggest its inclusion with the organisms of the TORCH acronym.

Infantile cataracts are rare diseases, with an incidence varying from 6.3–136 per 100,000 births (*1*–*5*). The etiology is diverse, including genetic disorders, metabolic diseases, or ocular infections. Intrauterine infectious causes are referred to by the TORCH acronym: toxoplasmosis, other infectious pathogens (such as HIV, syphilis, parvovirus B19/fifth disease, varicella/chickenpox, and Zika), rubella, cytomegalovirus, and herpes viruses (*6**,**7*).

Uveitis in children is even rarer; a recent study reported an incidence of 0.69/100,000 person-years in children from South Korea 0–6 years of age (*8*). Furthermore, uveitis manifests only exceptionally in the first months after birth. In a retrospective study performed in Finland, no child <1 year of age had uveitis (*9*). A study from India reported 15 cases of neonatal uveitis out of population of 1,450 premature infants. All those cases were of infectious etiology, with toxoplasma, rubella, cytomegalovirus, varicella zoster, *Mycobacterium tuberculosis*, other bacteria, and fungi as the causative organisms (*10*), illustrating that neonatal uveitis is usually infectious in nature and causative organisms are similar to those causing cataracts. Diagnostic tests are readily available for the most common of those pathogens, making diagnosis relatively straightforward.

In 2002, the first case of unilateral infantile cataracts with anterior uveitis caused by *Spiroplasma ixodetis* was published (*11*). In this article, we provide a review of the cases published before 2024 and evaluate an additional series of unpublished cases from different centers in Europe. We aim to describe a phenotype characterizing *Spiroplasma*-related cataracts and uveitis in infants through a review of the available literature and the newly published cases. In addition, we describe diagnostic steps and effective treatment options.

## Methods and Materials

After confirmation of a *S. ixodetis* diagnosis in a child from Romania and treated in Antwerp, Belgium, L.V.O. and M.T. contacted the authors of previously published cases and asked if they had encountered new patients since case publication. We also reached out to other centers that contacted the authors of the published cases for assistance in diagnosis and treatment of *S. ixodetis* in our patient. The resulting study group was named the *Spiroplasma* Infantile Cataract Group.

After initial contact through email, the group held a virtual meeting on June 1, 2023, to discuss the setup of the study and article. We determined patients were eligible for inclusion when cataracts were diagnosed and >1 of 3 diagnostic tests (16S RNA PCR, transmission electron microscopy [TEM], or culture) confirmed the presence of *S. ixodetis* in 1 or both eyes. Each center provided anonymized case reports for their cases with identifying parameters as limited as possible. We included patients through July 14, 2023. We combined the information from the new cases with information available from published cases (*12*–*16*). We then made separate tables summarizing the demographic data, diagnostic approach, and characteristics of the cataracts; the aspects of the uveitis; and the treatment details for each included case (Appendix 1 Tables 1, 2). We filled in the information on active cases after the tables were created on the basis of published reports.

Our study aim was 3-fold: first, to specify the characteristics of the clinical manifestations regarding the uveitis and the cataracts; second, to evaluate the different diagnostic modalities; and third, to compare the different treatment regimens used in the included cases. This research adhered to the tenets of the Declaration of Helsinki. Informed consent was obtained from the parents or guardians of every patient. The Ethical Committee of the Antwerp University Hospital considered this work exempt from formal review because of the observational nature of the research.

## Results

In addition to the 7 cases published to date (*11*–*14*), we have included information on 3 patients reported as 2 poster presentations (*15*,*16*) and 8 new patients. We have included a total of 18 children and 28 eyes from 8 countries in Europe (Germany, n = 6; France, n = 5; Austria, n = 2; Luxembourg, n = 1; the Netherlands, n = 1; Norway, n = 1; Romania, n = 1; and Switzerland, n = 1). Since finalization of this study, 1 additional case was published outside of this study group, reporting a similar clinical manifestation of uveitis, progressive cataracts, and ocular hypertension (*17*). 

We created a list of the clinical manifestations of *S. ixodetis* infection in the cases reported in this study (Table). In addition, we summarized the characteristics and demographics of the previously published cases and additional descriptions and figures of each (Appendix 1 Tables 1, 2; Appendix 2). 

**Table:** Common clinical manifestations documented in multicenter retrospective study of *Spiroplasma ixodetis* infantile cataract in 8 countries in Europe that should prompt the clinician to consider a diagnosis of *S. ixodetis*–related infantile cataract with uveitis*

Clinical manifestation in eyes (%)	Characteristics (%)	Additional comments
Lens opacity (100), can be highly asymmetric	Bilateral (55.6)	NA
	Unilateral (44.4)	NA
	Progressive over time (46.4)	NA
	White lens opacity (35.7)	NA
	Abnormal lens anatomy (25)	Fibrous plaque, fibrovascular membranes, improperly formed lens
Anterior uveitis (96.4), can be highly asymmetric	Extensive posterior synechiae (96.4)	May lead to the full seclusion of the pupil and increased intraocular pressure with enlarged corneal diameter
	Large endothelial precipitates (89.3)	Peculiar shape over the entire surface of the cornea
	Dilated immature iris vessels (71.4)	Can be adherent to the anterior lens capsule or to a pupillary membrane
	Pupillary membranes (50)	NA
	Involvement of the posterior segment (14.3)	Inflammation, retinal scars
Elevated intraocular pressure* (42.8)	Preoperative (14.3)	NA
	Postoperative (25)	NA
	Pre and postoperative (3.6)	NA
	Requiring glaucoma surgery (14.3)	NA

*We defined elevated intraocular pressure as the need for treatment for increased eye pressure on the basis of the treating physicians clinical judgement. NA, not applicable.

**Figure 1:**
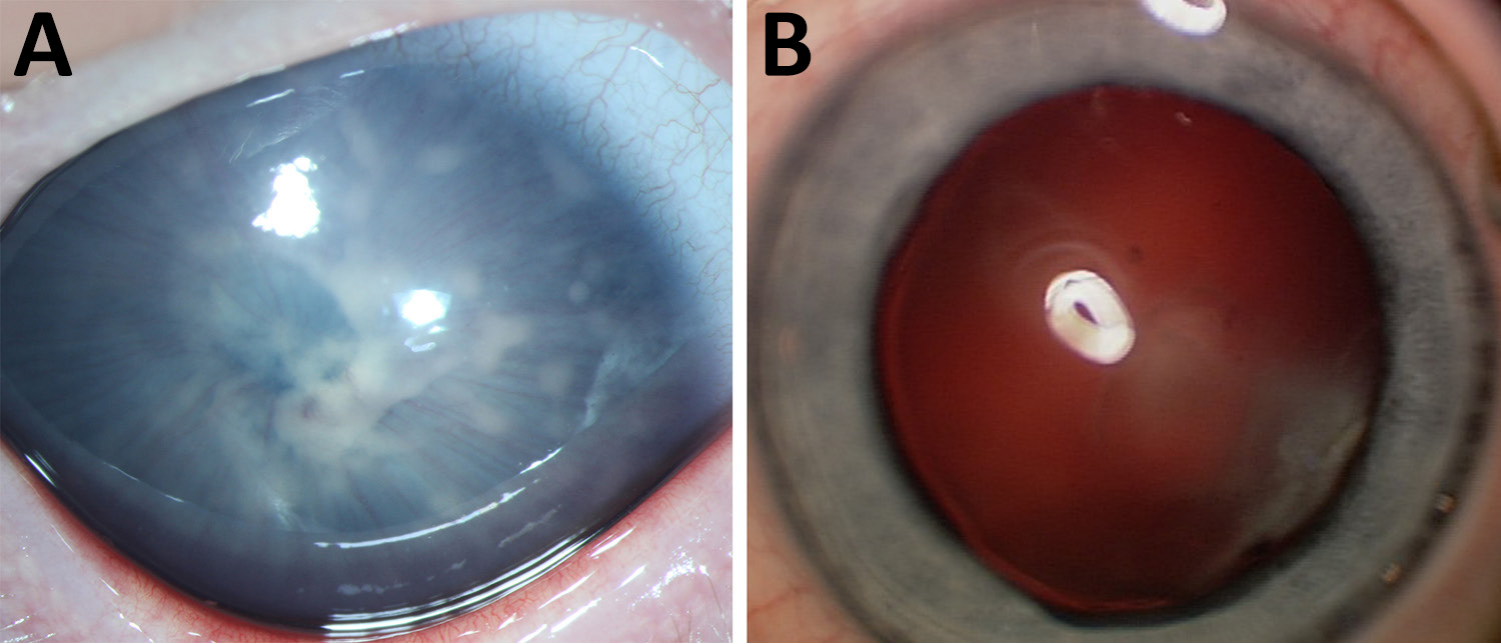
Spectrum of *Spiroplasma* spp.–caused eye disease documented in a multicenter retrospective study of *Spiroplasma ixodetis* infantile cataract in 8 countries in Europe. The study included a total of 18 children and 28 eyes. A) Preoperative image of patient 15, showing extensive endothelial precipitates and cataract and iris vascularization extending to the lens. B) Mild cataract in the left eye of patient 9 without apparent uveitis.

We found anterior uveitis in all patients (Figure 1, panel A). Anterior uveitis is classified as granulomatous or nongranulomatous depending on the clinical manifestation and does not refer to any histological aspects. A granulomatous uveitis is characterized by the presence of large endothelial precipitates or iris nodules. Of the 28 eyes from 18 patients included in the study, we found large endothelial precipitates in 25 (89.3%) eyes of 16 (88.9%) patients and iris nodules in 10 (35.7%) eyes of 8 (44.4%) patients. In patient 9, the uveitis affected only 1 eye, whereas the cataracts were bilateral, although less pronounced in the eye without uveitis (Figure 1, panel B). Patient 6 had vitreous infiltration, snowballs (cell aggregates in the vitreous), and some peripheral necrotic retinal infiltrates, and patient 10 demonstrated a clinical manifestation of endophthalmitis. Those patients’ clinical manifestations demonstrate uveitis can extend to the posterior segment. Patient 14 had unilateral cataracts with uveitis but bilateral macular scarring without a documented presence of posterior segment inflammation and a negative workup for other infectious causes. The relation of those scars to the inflammation is not clear.

Posterior synechiae were found in all eyes with uveitis (96.4%) and were often extensive, possibly leading to complete pupillary seclusion. In 14 (50%) eyes from 9 (50%) patients, a pupillary membrane was found. Both synechiae and pupillary membranes can lead to angle closure and a secondary increase of intraocular pressure (IOP), leading to an enlarged corneal diameter, which was seen in patients 5, 7, and 8. Twelve (42.8%) eyes from 9 (50%) patients had an increased IOP. Four (14.3%) eyes from 4 (22.2%) patients had increased IOP before cataract surgery, and increased IOP developed in 7 (25%) eyes from 6 (33.3%) patients after the initial cataract surgery. One (3.6%) eye from 1 (6.3%) patient had increased IOP before and after cataract surgery. Two patients (patients 8 and 18) had 1 eye with increased IOP before surgery and developed increased IOP in the other eye after surgery.

Of the 12 eyes with ocular hypertension, 4 needed glaucoma surgery (14.3% of all eyes): patient 8 (right eye) underwent a trabeculotomy, patient 14 (right eye) received a glaucoma drainage device, and patient 16 (left eye) underwent trabeculectomy with mitomycin C. Patient 18 (left eye) underwent cyclophotocoagulation because of severe scarring of the trabecular meshwork and continued to require topical IOP lowering medication. Furthermore, 20 (71.4%) eyes from 14 (77.8%) patients demonstrated severely dilated iris vessels adherent to the lens (Appendix 1 Tables 1, 2).

Eight (44.4%) infants had unilateral cataracts, of whom 1 showed bilateral macular scars without the presence of uveitis. Bilateral cataracts manifested in 10 (55.6%) infants; patient 9 was affected by cataracts in both eyes but uveitis only in the right eye.

The cataracts were variable in clinical manifestation. In 10 (35.7%) eyes, the cataracts manifested as a white cataract within the first weeks of life. In 13 (46.4%) eyes, we found the cataracts got denser during the first months of life, as described previously (*11*). No progression was reported in 5 (17.9%) eyes.

Abnormal lens anatomy was found in 7 (25%) eyes from 5 (27.8%) patients. The abnormal anatomy found included fibrotic subcapsular membranes (patients 8 and 14), persistent fibrovascular membranes (patient 17), small and deformed lens (patients 8 and 15), and lens subluxation (patient 18).

**Figure 2:**
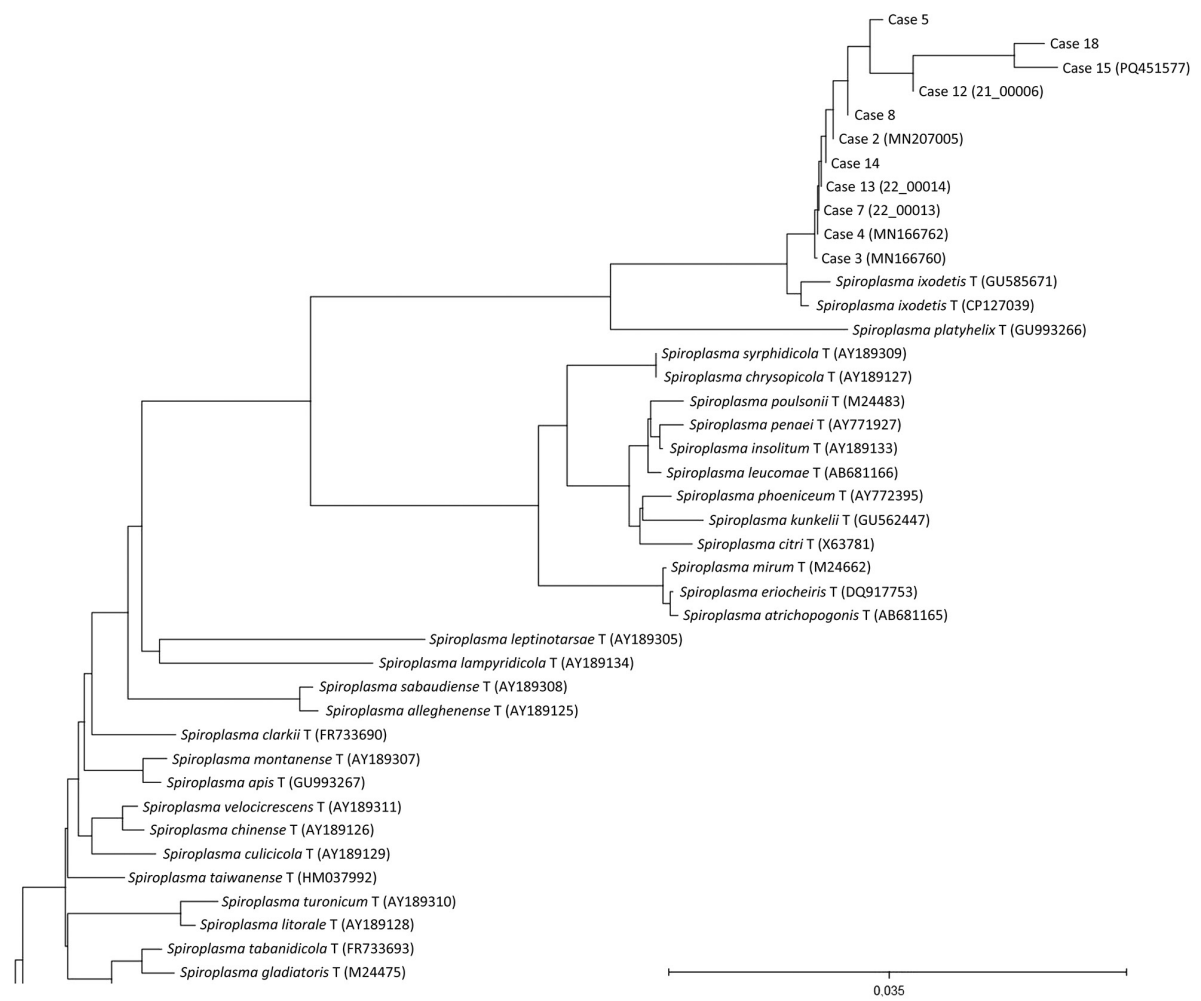
Neighbor-joining unrooted tree based on *rrs* gene sequences recovered from a multicenter retrospective study of *Spiroplasma ixodetis* infantile cataract in 8 countries in Europe. The study included a total of 18 children and 28 eyes. Numbers in parentheses are GenBank accession numbers. Scale bar represents substitutions per nucleotide position.

There is currently no standardized technique to diagnose *Spiroplasma* infection. 16S-rRNA PCR was performed on lens fiber material from the patients reported in this study. The PCR testing generated a positive result in 25 (89.3%) eyes. We created a phylogenetic tree with the 16S sequences (Figure 2); the available sequences were uploaded to the GenBank database. TEM was performed on lens material in 10 eyes from 7 patients and identified *Spiroplasma* spp. within lens fibers in 9 (90%) of the eyes tested. Culture was attempted on lens material of 8 eyes from 6 patients and *S. ixodetis* was cultured from 7 (87.5%) of those eyes (*14*).

Of the 18 patients, 77.8% received topical corticosteroids before surgery, and all patients had topical corticosteroids in the postoperative period. In addition, 5 (27.8%) patients received systemic steroids to control the ocular inflammation. Systemic antimicrobial treatment was used in 17 (94.4%) patients. In 16 (88.9%) patients, the antimicrobial drug was a macrolide (erythromycin, azithromycin, clarithromycin, or josamycin). The remaining patient (patient 5) received a systemic antimicrobial drug, but we could not determine which type.

## Discussion

We describe 28 eyes in 18 infants from 8 countries in Europe that had cataracts and intraocular inflammation caused by *S. ixodetis*. Three modes of diagnosis were used: 16S-rRNA PCR on aspirated lens tissue, TEM on lens tissue, and culture on aspirated lens material (Mycoplasma A7 agar medium [ELITechGroup]). All 3 tests seemed equally likely to generate a positive result in the affected eyes. PCR was positive in 89.3% of eyes (all eyes were tested), TEM was positive in 90% of 10 eyes tested, and culture was positive in 87.5% of 8 eyes where culture was attempted. The positive cultures are an additional requirement of Koch’s postulates to confirm *S. ixodetis* as the cause of this clinical entity. Lens tissue was obtained by simple aspiration with either a syringe or a vitrector. Patients from cases 9 and 10 demonstrate that PCR can be negative but TEM can still confirm the diagnosis; therefore, we recommend using each of the available diagnostic paths in cases with a high clinical suspicion.

In several of the patients, aqueous humor samples were analyzed by 16S-rRNA PCR. This testing yielded a positive result only for case 11, in which the aqueous humor sample was obtained after lensectomy and could have been contaminated with *Spiroplasma* spp. DNA from inside the lens. *Spiroplasma* spp. most likely cannot survive in the eye outside of the lens and needs the presence of cells for survival. The refreshing of the aqueous humor within the eye could play an additional role in the absence of positive PCR results. This potential role highlights the need to perform PCR on lens tissue obtained during cataract surgery to confirm the diagnosis.

Cataracts were bilateral in 55.6%, unilateral in 44.4%, and progressive in 46.4% of patients. All patients had a granulomatous uveitis in >1 eye, with thick endothelial precipitates scattered over the entire endothelium. Similar deposits were seen on the iris. The uveitis can be highly asymmetric. Diagnosis of a very limited uveitis in a newborn can be challenging and could easily remain unnoticed.

In the left eye of patient 9, no uveitis was found, and the cataracts were very limited, although they were progressive over time. PCR and TEM results were positive in the eye with uveitis (right eye) but negative in the left eye. This positivity might indicate the load of *Spiroplasma* spp. was lower in the eye with mild cataracts and no detectable uveitis and that the inoculum of *Spiroplasma* spp. has a role in the pathophysiology. Furthermore, the examinations are conducted on only a portion of the removed lens tissue, which can leave the *Spiroplasma* spp. undetected. The presence of minor lens changes could disturb the normal metabolism of the lens, causing the cataracts to progress further over time. This progression possibly happens in the absence of the *Spiroplasma* spp. that triggered the cascade. The cataract with negative PCR result illustrates that *Spiroplasma* spp. could cause an isolated mild cataract without signs of inflammation and may escape detection by PCR and TEM.

Cataract progression was seen in 46.4% (n = 13) eyes before surgery, even after reducing inflammation, as previously described (*11*,*16*). Several factors could be involved in this progression, and they are not mutually exclusive. Topical steroids, when used for several months, can cause progression of cataracts, as can the ongoing anterior chamber inflammation. The clinical picture of cataracts in those cases does not fit the manifestation of a purely corticosteroid-induced cataract, and cataract was documented before the start of steroids in most cases. Extensive fibrosis of the lens capsule was noted in 3 patients (patients 8, 14, and 17), and the lens was deformed in 3 patients (patients 8, 15, and 18) with abnormal or absent zonules. This finding might indicate that the infection with *Spiroplasma* spp. can interfere with normal lens development. Progression of the cataract might be because of poor penetration of the antimicrobial in the lens material, enabling *Spiroplasma* spp. to trigger the capsular lens epithelial cells to transform into fibroblasts and lose their transparency.

The timing of surgery for infants with cataracts is critical to avoid amblyopia. A balance between early surgery to improve visual prognosis and reduction of inflammation before surgery needs to be sought. We recommend starting a systemic antimicrobial drug treatment as soon as *Spiroplasma* spp. is suspected and adding an antiinflammatory treatment guided by the amount of intraocular inflammation. In this review, treatment regimens varied between patients, but most received a perioperative systemic macrolide antimicrobial in combination with topical or systemic steroids. The inflammation resolved rapidly once treatment was started. The patient in case 3 did not receive systemic antimicrobial drugs, only topical tobramycin in the first postoperative month. In that patient, there were no recurrences in the 18 months after surgery. The lens removal could be part of the treatment because it is possible *Spiroplasma* spp. cannot survive in the eye after lens removal.

Intraocular lens (IOL) implantation is considered controversial both in young children and in eyes with inflammation. In patients 8 and 10, an IOL was implanted after the bag-in-the-lens technique. In our experience implanting the lens, we saw very little postoperative inflammation in patients with uveitis, even at a young age (*18*,*19*). However, the threshold for implantation of an IOL in those children should be very high, no classic in-the-bag approach should be used, and aphakia is preferred when there is any doubt regarding activity of inflammation.

Reactivation of uveitis has not been reported in any of the previously published cases. However, 2 of the patients reported in this article suffered a reoccurrence after initial control of the inflammation. Those relapses were treated with a combination of a repeated course of systemic macrolide antimicrobials and a low dose of topical steroids, which was slowly tapered.

A strength of this study is that we provide additional information on many newborns with *Spiroplasma-*induced cataracts and uveitis in a multicentric cooperation. This additional information adds to the known information on the topic, therefore strengthening the generalizability of the data.

A limitation of this study is that we cannot give a clear mechanism of the infection with *Spiroplasma* spp., but our findings could serve as a basis for further research to enlighten this process. A standardization of the diagnostic approach can make diagnosis easier and will help in giving those children the appropriate treatment.

The genus *Spiroplasma* belongs to the class Mollicutes, which contains only bacteria without a cell wall. *Spiroplasma* spp. can be found on plants, in insects, or crustaceans (*20*). *Spiroplasma* spp. are symbionts of ticks and are transmitted vertically from the female tick to her eggs (*21*). *S. ixodetis* was first described in Western blacklegged ticks (*Ixodes pacificus*) in Oregon, USA (*22*), and has since been identified in other tick species, such as *I. ricinus* and *Dermacentor marginatus* (*23*). Several studies have found a heterogeneous geographic distribution of the presence of *Spiroplasma* spp. in harvested ticks (*24*–*26*).

*Spiroplasma* spp. is not generally considered a human pathogen, but several reports of human disease caused by *Spiroplasma* spp. have been documented (*11*–*15*,*27*–*30*). The first reported human infection with *Spiroplasma* spp. in a premature child with unilateral cataract and uveitis occurred within a prospective study examining lens material from a series of infant cataracts that were not hereditary in origin (*11*). Since then, further cases of coincident progressive cataract and granulomatous uveitis because of infection with *Spiroplasma* spp. in neonates have been published (*12*–*17*). We identified a small number of reports on systemic infections with *Spiroplasma* spp. occurring in immunocompromised patients (*27*,*29*,*30*). One case in an elderly woman (*28*) led to the suggestion of advanced age as a possible cause of relative immunosuppression. In addition, 1 report identified *Spiroplasma* spp. in a series of persistent root canal infections (*31*).

A derivative of a related species, *S. mirum*, known as suckling mouse cataract agent (SMCA), has been used in experimental eye models. In those models, injection of SMCA into the brain of newborn rodents caused inflammatory eye disease, microphthalmia, and structural defects of cornea, lens, and retina. In adult animals, no disease could be induced (*32*–*34*). Those findings confirm the preference for the eye as a target organ and agree with the reports of uveitis and cataracts in newborns caused by *S. ixodetis*.

The exact mode of infection in the patients reviewed in this article is unclear. In the first published case and in cases 9 and 10, a maternal infectious disease was noted during the pregnancy. In patient 9, a *Mycoplasma* infection was suspected. *Mycoplasma* also belongs to the class Mollicutes and therefore could have been a misdiagnosis of an infection with a relative of *Spiroplasma* spp. In patients 8 and 11, there was a positive history of a tick bite during the pregnancy, as well as a wasp sting in case 11. That history demonstrates 2 possible modes of maternal infection: either community-acquired or by an arthropod vector (most likely a tick), but other modes of infection could still be possible. Those modes of infection may not be mutually exclusive and could lead to a possibly asymptomatic or mild maternal infection.

Because infection is possibly transplacental (*11*), both eyes would be equally likely to be affected. However, of note, the manifestations ranged from unilateral cases (44.4%) to asymmetric and bilateral cases (55.6%) in both cataracts and uveitis. We also found a case of a child from a diamniotic, dichorionic twin in which the sibling was unaffected (patient 18) (Appendix 2).

Because *S. ixodetis* can be cultured from lens tissue of affected infants and positive PCR results are similarly found in the lens tissue itself, *S. ixodetis* might be contained inside the lens, within the barrier formed by the lens capsule. TEM clearly shows the intracellular location within lens fibers (*11*,*16*). This containment could mean *S. ixodetis* was already present before the closure of the lens vesicle (*35*), as described previously (*36*). However, in animal studies (*32*–*34*), pathological changes in the lens are seen in rabbits inoculated with SMCA within 48 hours of birth, which indicates SMCA can penetrate the lens capsule of an immature lens after birth. Research on the involvement of *S. mirum* in transmissible spongiform encephalopathies has shown *Spiroplasma* spp. use curli-like fibers to drill into the cell wall (*37*). There may be a similar pathway enabling *S. ixodetis* to perforate the lens capsule. Because *Spiroplasma* spp. have been proven to cause cataracts in both newborn humans (*11*–*17*) and newborn animals (*32*–*34*), it is possible the ability to invade the lens is limited to a certain period, probably related to the immaturity and thickness of the lens capsule at the time of infection (*35*). Both pathways of infection, either before closure of the lens vesicle or by penetration of the immature capsule, are not mutually exclusive.

Factors such as timing of the infection, genetic susceptibility, the inoculum of *S. ixodetis*, host immune responses, or differences in the local microenvironment could influence the development and severity of the disease. To better study those factors and further characterize the disease, we are setting up a prospective study with a standardized diagnostic and treatment approach. This prospective study might generate more robust data on the performance of the different diagnostic modalities and can help in developing a targeted quantitative PCR, which would make diagnosis easier.

Because of the rarity of these manifestations, we suggest considering *S. ixodetis* along with the organisms implied in the TORCH acronym in the diagnostic workup of cataracts and uveitis in infants. Analysis of lens material with 16S-rRNA PCR, culture, and TEM is crucial to confirm the diagnosis.

Appendix 1 Additional tables with information about multicenter retrospective study of *Spiroplasma ixodetis* infantile cataract in 8 countries in Europe.

Appendix 2 Additional information, cases, and figures from a multicenter retrospective study of *Spiroplasma ixodetis* infantile cataract in 8 countries in Europe.
