# Supplementary material for: Multicenter Retrospective Study of Spiroplasma ixodetis Infantile Cataract in 8 Countries in Europe
Source: Emerg Infect Dis. 2025 Jun;31(6):1081–9. doi: 10.3201/eid3106.240954 (PMC12123929; doi:10.3201/eid3106.240954)
Supplement: Appendix 2 — Additional information, cases, and figures from a multicenter retrospective study of Spiroplasma ixodetis infantile cataract in 8 countries in Europe. [file 24-0954-Techapp-s2.pdf]

Article DOI: https://doi.org/10.3201/eid3106.240954

EID cannot ensure accessibility for supplementary materials supplied by authors. Readers who have difficulty accessing supplementary content should contact the authors for assistance.

Multicenter Retrospective Study of *Spiroplasma ixodetis* Infantile Cataract in 8 Countries in Europe

Appendix 2.

This appendix provides more elaborate information on the previously unpublished cases. The numbering starts with case 8, as the previous 7 cases have been published in the literature (*1*–*4*).

Case 8

A full-term born baby girl presented at the age of two weeks to the Emergency children's hospital "Marie S Curie" in Bucharest (Romania) in 2022. The family lives in a rural area in Romania and the mother mentioned having had a tick bite around the sixth month of pregnancy with an inflammatory reaction around the site of the bite.

The baby suffered from a bilateral anterior uveitis with an asymmetrical cataract, the left eye being the most affected. Both eyes showed large endothelial precipitates, extensive posterior synechiae and a pupillary membrane with iris vascularization extending to the anterior lens capsule (Figure 1A, 1B). The intraocular pressure (IOP) was high on the left eye due to angle closure secondary to anterior synechiae. An enlarged corneal diameter was evident and secondary to the increased intraocular pressure.

Treatment was started with topical dexamethasone, tropicamide, and dorzolamide/timolol combination. Furthermore, IV Methylprednisolone and Clindamycin (lincosamide antibiotic) was started. Although the inflammation rapidly decreased, Clindamycin was switched to Josamycin (macrolide antibiotic) as suggested by the paper by Matet et al (*2*). The eye pressure

normalized to 16 mmHg in the left eye and the endothelial precipitates disappeared (Figure 2). The child was then referred to undergo cataract surgery.

Surgery was performed at the age of 12 weeks in the left eye and one week later in the right eye. The inflammation had quietened down at the time of surgery. Hence, we decided to implant a lens following the bag-in-the-lens technique with an intraocular lens (IOL) type Morcher 89A (Morcher, Germany). The anterior lens capsule in the left eye was very opacified in the center with a thick fibrotic plaque underneath. During the surgery of the right eye, we observed a large peripheral fibrotic membrane at the superior part of the lens. In this eye, this seemed to have prevented the completion of the capsular bag and the corresponding zonules resulting in an incomplete crystalline lens formation superiorly (Figure 3).

On the right eye, 2 samples of manually aspirated lens material showed a positive PCR result for *Spiroplasma ixodetis* on 16S-rRNA PCR. On the left eye the fibrous plaque and one of the two lens samples were positive. Transmission electron microscopy (TEM) and culture were not performed in this case.

Postoperatively, we used fixed combination eye drops of Tobramycin/ dexamethasone 6 times per day for one week, then 3 times per day for another week. Additionally, we used diclofenac eye drops 4 times daily for one month. The Dorzolamide/timolol combination was continued in the left eye for the first postoperative month.

One month after surgery, the eyes were quiet, with clear IOLs and further normal anterior chambers. There was one anterior synechia in the angle at the incision site of the left eye as a complication of the surgery. The intraocular pressure was 17 mmHg in the right eye and 9 mmHg in the left eye. The optic disc was normal but with some peripapillary atrophy or scarring in the left eye. Furthermore, the retinal vessels in the left eye were notably narrower than in the right eye. This might indicate some past inflammatory involvement of the posterior segment.

Glasses were prescribed to correct the remaining refractive error and occlusion therapy was started to treat amblyopia in the right eye.

Three months after the surgery there was a slight reactivation of anterior uveitis with endothelial precipitates, for which topical steroids were started again and tapered slowly. Due to

a persistence of the inflammation, Clarithromycin (macrolide antibiotic) was added to the regimen. The uveitis remained quiet hereafter.

Eight months after the surgery for the right eye, the intraocular pressure on this eye increased to 26 mmHg. Topical steroids were stopped but the IOP did not lower. Hence, a 270° trabeculotomy was performed one month later, resulting in a pressure of 14 mmHg one week after the procedure.

Case 9

A 6 month-old infant, born at term, presented in 2009 to the Department of Ophthalmology of the Justus-Liebig-University in Giessen (Germany), with bilateral cataract and heterochromia associated with severe anterior uveitis in the right eye and no further history of infection. (Figure 4A, 4B). The mother had experienced an upper respiratory tract infection during pregnancy. Retrospective evaluation of a baby photo taken at age 4 weeks clearly showed that the red reflex was already duller in the right eye (Figure 4E).

Anti-inflammatory therapy was effective to reduce the uveitis. (Figure 4C, 4D) Cataract surgery was performed at age 31 and 35 weeks by pars plana-pars plicata lensectomy combined with anterior vitrectomy.

TEM confirmed *Spiroplasma* species in the lens material collected at the time of surgery (Figure 6A, 6B). The diagnosis was further strengthened with PCR on the lens material of the right eye, where a positive PCR product (474bp) was acquired. BLAST analysis revealed a 99.7% match with *Spiroplasma* species 16S-rRNA sequences (*5*) (Figure 6E). However, further attempts to sequence larger parts of the 16S-rRNA with different primer sets remained unsuccessful. The lens material of the left eye showed a negative PCR result for *Spiroplasma*. The data was considered convincing proof for *Spiroplasma* species as the causative agent. *Spiroplasma* species could not be detected by PCR in serum or aqueous humor.

This case has been presented as a poster on the 36th Annual Meeting of the European Paediatric Ophthalmological Society in Bad Nauheim, Germany in 2010 (*6*).

Case 10

A preterm baby, born in 2010 at 26 weeks 5 days with a birthweight of 490g and a history of intravitreal bevacizumab for acute retinopathy of prematurity (ROP) at 35+1 weeks post-menstrual age (PMA), bronchopulmonary dysplasia, pulmonary artery hypertension with respiratory insufficiency, prolonged cardio-pulmonal resuscitation at 33+4 and 51+4 weeks PMA, hernia, hepatopathy with cholestasis of unclear origin and epilepsy, cerebral palsy, post-hypoxic cerebral lesions and psychomotor retardation presented to the Department of Ophthalmology of the Justus-Liebig-University in Giessen (Germany). In this case, an untreated amnion infection was assumed in the mother, who had a history of diabetes and nicotine abuse. This child developed a mature cataract at age 8 months following an endogenous endophthalmitis of unknown origin (Figure 5). The cataract was bilateral and progressive yet asymmetric (left eye > right), and consisted of an anterior opacity in the fetal nucleus.

Cataract surgery with an anterior chamber approach was chosen in this case, with implantation of an IOL type Morcher 89A (Morcher, Germany) at age 12 and 21 months, respectively.

TEM confirmed *Spiroplasma* species in the lens material collected at the surgery in this case as well (Figure 6C, 6D). Both probes of this case were negative on PCR for *Spiroplasma*, although the entire lensectomy material was used. Neither could *Spiroplasma* species be detected by PCR in serum or aqueous humor. As in the report of the first case (*1*) the TEM-profiles of the wall-less *Spiroplasma* exhibited typical round, curved, or helical shapes (Figure 6C, 6D). Also noted were spherical or irregular vesicular organisms. The size of the profiles varied from 60 nm to 120 nm in diameter. The internal aspect of the organisms often had a filamentous appearance corresponding most to the characteristic *Spiroplasma* associated fibrils (SpFs) previously described by Bastian et al. (*7*)

This case has been presented as a poster on the 36th Annual Meeting of the European Paediatric Ophthalmological Society in Bad Nauheim, Germany in 2010 (*6*).

Case 11

A female infant was referred to the Eye center of the university of Freiburg (Germany), three days after full-term birth. Bilateral cataracts were noticed in the pediatric screening (“U2-Vorsorgeuntersuchung”). Family history for hereditary cataracts was negative and pregnancy was inconspicuous. However, the mother did remember being bitten by a tick in the 7th month of pregnancy. She was also stung by a wasp during her pregnancy at a time she could not remember. She did not experience any medical problems after the tick bite nor after the wasp sting. At initial presentation, we found diffuse white cataracts without any signs of ocular inflammation in both eyes. The fundus was obscured bilaterally. Ultrasonography showed a normal posterior eye segment in both eyes. IOP was normal. Ten weeks later, we performed a lensectomy. At this time, we saw a progressive opacification of the lens as well as confluent corneal endothelial precipitates and posterior synechiae as signs of anterior uveitis in both eyes (Figure 7). There was no sign of inflammation in the vitreous or retina.

Dexamethasone and cefuroxime (cephalosporin antibiotic) (each 0.1 ml of dexamethasone 4.44 mg/ml and cefuroxime 20 mg/ml) were injected into the anterior chamber. Betamethasone (in total 0.1 ml of betamethasone acetate 3.0 mg/ml and betamethasone sodium phosphate 3.95 mg/ml) was injected under the conjunctiva.

PCR (comprising Toxoplasma gondii qPCR, Staphylococcus aureus qPCR, eubacterial PCR, Mycoplasma multiplex n-PCR, CMV-PCR, HSV-1/2-PCR and VZV-PCR) of lensectomy probes and anterior chamber fluid (obtained after lensectomy) revealed *Spiroplasma ixodetis*.

Postoperatively, the patient received tapering topical steroids, tapering topical antibiotic eye ointment (Ofloxacin 3 mg/g) and intravenous azithromycin (macrolide antibiotic) once daily for three days. Contact lenses were fitted.

Case 12

In 2020, a 10-day-old boy was referred to the Fondation Rothschild in Paris (France) for unilateral leukocoria, which his parents had noticed at birth. He is the second child of non-consanguineous parents, with a history of congenital glaucoma in a paternal uncle. He was born

after a normal full-term pregnancy, without delivery complications, and without maternal seroconversion for toxoplasmosis, rubella, herpes simplex viruses 1 and 2, or cytomegalovirus.

Examination of the right eye showed unilateral right anterior uveitis with large endothelial precipitates, endothelial and iris nodules, posterior synechiae, immature dilated iris vessels, and cataract. The fundus could not be seen, and the right eye was not microphthalmic. Results of the physical examination were unremarkable.

Treatment was initiated with topical dexamethasone (4 drops/day with progressive tapering), topical atropine (0.3%, 2 drops/day), and oral josamycin (125 mg twice daily). Four days after the first visit, the boy underwent an ophthalmologic examination under anesthesia (Figure 8). The corneal diameter was similar for both eyes, but pachymetry was increased on the right side (733 µm vs. 630 µm), without associated ocular hypertonia. Axial length was within the normal age range on both sides. Ocular ultrasonography under anesthesia ruled out retinoblastoma and did not show signs of posterior segment inflammation. The anterior chamber inflammation decreased dramatically (Figure 9), and cataract surgery without intraocular lens implantation was performed 6 weeks later when the child was 2 months old.

We conducted microbiological investigations of lens aspirates from the right eye, including bacteriologic and mycologic cultures, as well as 16S-rRNA-based PCR for bacterial identification. The A7 agar medium (ELITechGroup, Signes, France) allowed to culture *Spiroplasma*. From the fresh crystalline lens sample, the colonies began to grow after 4 to 10 days at 30°C. On A7 agar, the colonies were only visible at a x40 magnification of a photonic microscopy. The tiny colonies were embedded in the agar medium. Three months after a subculture, the colonies were bigger and took on the shape of a "fried-egg" (Figure 10). 16S-rRNA-based PCR of the bacteria rrs gene performed on crystalline lens samples (Case 12, 21_00006) identified *Spiroplasma ixodetis* (Figure 13).

Case 13

In 2022, a 16-day old girl was referred to the Fondation Rothschild in Paris (France) because of unilateral leukocoria associated with anterior uveitis. She was the first child of non-consanguineous parents, born after normal full-term pregnancy, without delivery complications

and without maternal seroconversion for toxoplasmosis, rubella, herpes simplex viruses 1 and 2, or cytomegalovirus.

The examination of the right eye showed unilateral right anterior uveitis with large endothelial precipitates, endothelial and iris nodules, posterior synechiae, immature dilated iris vessels, and cataract. The fundus could not be seen and the eye was not microphthalmic. Physical examination results were unremarkable.

Treatment with topical dexamethasone (6 drops/day with progressive tapering), topical atropine (0.3%, 2 drops/day), topical intraocular pressure-lowering treatment and oral josamycin (125mg twice daily) was initiated. Anterior chamber inflammation decreased significantly and cataract surgery without intraocular lens implantation was performed 5 weeks later when the child was 2 months old.

16S rRNA-based PCR analysis of the bacterial rrs gene from crystalline lens samples (Case 13, 21_00014) identified *Spiroplasma ixodetis*, and culture also showed growth of *Spiroplasma ixodetis* (Figure 12).

She experienced a relapse one month after the surgery, noted during the removal of sutures under general anesthesia (Figure 11). At the time, she was receiving one drop per day of dexamethasone combined with polymyxin b sulfate and neomycin sulfate (Maxidrol®). Treatment was increased to four drops per day in combination with oral josamycin (125mg 2x/d). Inflammation decreased within 15 days, and a slower tapering over six weeks allowed for recovery. A second relapse was observed during the follow-up, again when the topical corticosteroids were reduced to one drop per day. Topical corticosteroids were increased to 6 drops/day with a slower tapering during 3 months, allowing the inflammation to be controlled at the age of 9 months. She also presented with ocular hypertension, for which she received beta-blocker eye drops.

Case 14

A girl, born at 37 weeks gestation, had a previous hospital admission elsewhere due to a respiratory infection, treated with oxygen and nasal decongestion but no antibiotics. The mother had pre-eclampsia during the pregnancy for which she received anti-hypertensive medication. Furthermore, she mentioned a COVID infection during week 7 of the pregnancy and had no

recollection of tick bites. The father is a heterozygote carrier for CLN3, USH2A. Evaluation of the child by the pediatrician and medical geneticist revealed a normal hearing test, no genetic defects and a negative TORCHES-screening.

At the age of 11 weeks she presented to the Amsterdam University Medical Centers (The Netherlands) with absent visual contact, without nystagmus. The right eye showed an anterior uveitis with mutton fat corneal endothelial precipitates, differing in size and spread over the entire cornea, prominent iris vessels, and a small non-reactive pupil with posterior synechiae. Furthermore there was a cataract with an impaired red reflex, enabling only a faint impression of the optic disc. The anterior chamber was deep and there was no redness of the eye. The left eye showed an entirely quiet anterior segment and vitreous but a large atrophic macular scar (Figure 14). Ultrasonography of the right eye revealed no signs of vitreous or retinal pathology. The IOP measured by rebound tonometry (iCare) was normal (9 mmHg RE, 8 mmHg LE). Despite treatment with prednisolone eye drops (3 times daily) and cyclopentolate 0.5% drops (twice daily), the mutton fat precipitates persisted.

The density of the cataract increased and a pupillary membrane with granuloma and posterior synechiae formed, causing the iris to bulge forward somewhat. Rebound tonometry at this time measured 16 mmHg in both eyes. An anterior chamber puncture was performed for PCR and Goldmann-Witmer testing for a number of known pathogens (herpes simplex 1 and 2, varicella zoster, cytomegalovirus, toxoplasma, rubella), but remained negative.

A cataract extraction was performed in the right eye by an anterior approach using limbal incisions. This was combined with a repeat aqueous tap and aspiration of lens material with the vitrectome for microbiological examination and panbacterial 16S-rRNA based PCR. The eye showed iris hyperemia, vasculature over the cataract and extensive fibrosis of the anterior and posterior capsule and lens. Iris hooks and the vitrectome were used to extend the pupil opening and an anterior vitrectomy was performed.

Subconjunctival steroids (betamethasone) and intracameral antibiotics (cefuroxime) were administered. Topical steroids and antibiotics (tobramycin/ dexamethasone combination (Tobradex®) 4 times daily) as well as cyclopentolate 0.5% twice daily was administered. Ten days later an anterior bowing of the iris and near-total seclusion of the pupil was present due to the formation of a pupillary membrane, with an IOP of 14 mmHg. A surgical dissection of the

pupillary membrane, sphincterotomies and peripheral iridectomy were performed using the vitrectome. Fundoscopy revealed a large macular scar, identical to the one in the left eye.

At the 1 month follow-up the eye was quiet with a clear visual axis, some subconjunctival steroid deposit visible, and a raised intraocular pressure (20 mmHg compared to 10 mmHg in the unaffected left eye). 16S-rRNA PCR sequencing detected *Spiroplasma* from the lens aspirate. All other examinations were negative for TORCHES.

The patient was then treated with oral azithromycin 40 mg/ml, 52 mg once daily for 3 weeks. Four months after the cataract removal, glaucoma surgery was performed (Paul glaucoma implant) due to intraocular pressure up to 46 mmHg in spite of topical treatment with timolol and dorzolamide twice daily. At the most recent follow-up (three months after the glaucoma surgery), the IOP was good (8 mmHg RE, 12 mmHg LE) and the anterior segment was quiet in both eyes.

Case 15

In December 2019, a 3-day-old female infant was referred to the clinic at Oslo University Hospital (Norway) with a unilateral anterior uveitis in the right eye. Clinical presentation showed pronounced anterior uveitis with atypical, multiple mutton-fat precipitates in different shapes and sizes, spread in the anterior chamber, deposited both on the corneal endothelium and on the iris. There were posterior synechiae and atrophy of the iris, hyperemia of the iris, and a persistent pupillary membrane. The lens was initially clear, but was smaller than usual and deformed. There were no signs of posterior uveitis. (Figure 15 A-C) An increased introacular pressure was noticed from 3 weeks of age and treatment with timolol 1mg/ml eye drops was started. The other eye showed no signs of uveitis, but there was a subtle, insignificant cataract with tiny small particles centrally in the nucleus.

The infant was otherwise healthy and born at full term. An extensive diagnostic work-up showed no underlying systemic disease. The mother had suffered bronchitis in the third trimester of the pregnancy, with a full recovery. The father had suffered acute sarcoidosis four years earlier with a full recovery. Both parents and siblings were otherwise healthy. The family lives on the coast of the south-eastern part of Norway, an area known as a common habitat for ticks. It is unknown whether ticks *(Ixodes ricinus*) in this area harbor *Spiroplasma ixodetis*. However,

infection caused by *Spiroplasma ixodetis* has been reported after tick exposure in Sweden (*8*) The mother has no knowledge of having obtained tick bite during pregnancy.

Topical and systemic steroids (prednisolone 1,7 mg/kg for 3 weeks) were started to control the ocular inflammation. Because of suspicion of *Spiroplasma*, systemic Erythromycin (macrolide antibiotic) was started and continued for 3 weeks. After one week of treatment, the eye showed very little signs of uveitis. By this time, a dense cataract had developed and the decision was made to proceed to surgery. Cataract surgery was performed at 11 weeks of age. During the procedure we obtained aqueous humor, lens capsule of both anterior and posterior rhexis and aspirate of the lens material for microbiological examination. The eye was left aphakic.

The PCR analysis of lens material detected *Spiroplasma* species with 98.0% similarity to *Spiroplasma ixodetis*.

Treatment with macrolides was continued for 5 additional days (total treatment duration of 4 weeks) and systemic steroids were continued at the preoperative dose for one week and then tapered gradually over 6 weeks in the postoperative phase. After discontinuation of all treatment, the uveitis resolved completely and the condition was considered to be cured, with no sign of relapse over 3 years of follow-up. The IOP remained elevated after the surgery and the timolol eye drops were supplemented 4 weeks after the surgery with brinzolamide eye drops twice daily. With this topical treatment, the eye pressure has been well controlled.

Case 16

In 2012, a 3-month-old female full-term child with congenital clump feet was diagnosed with a mature unilateral cataract in the left eye at the University Hospital Vienna, Austria. In the fellow eye, the lens was initially clear, showing no abnormalities nor inflammatory signs. The child's family was living in a rural surrounding. No abnormalities during pregnancy or delivery could be documented. At the age of 3 months, we performed a lensectomy under general anesthesia. The vessels of the iris were severely dilated with adhesive growth to the anterior lens capsule. We used an anterior approach by two 23g paracenteses, combined with anterior vitrectomy and removal of the massive membranes and fibrovascular tissue. The eye was left aphakic.

The postoperative topical treatment consisted of prednisolone acetate 10 mg/ml eye drops 6 times per day, ofloxacine 3 mg/ml eye drops 4 times per day and bromfenac 0.9 mg/ml eye drops 3 times per day. There was no inflammatory relapse in the postoperative course.

At the age of 5 months, the pediatricians diagnosed a dilatative cardiomyopathy of unknown origin with severely impaired myocardial function. At the age of 6 months, a severe anterior uveitis was found in the right eye, which was primarily apparently unaffected. The optic axis was obscured by a cataract and thick fibrovascular tissue, and the iris showed massive dilation of vessels comparable to the left eye (Figure 16). Anti-inflammatory local therapy with corticoid eye drops was not sufficient to suppress the inflammation. The child underwent extensive evaluation by the pediatric department; however, no underlying disease or infection could be proven by serological tests. Subsequently, the child was administered systemic antimicrobial treatment with erythromycin. After 10 days of treatment, the inflammation of the right eye decreased and myocardial function improved. After the right eye was stabilized with concern to the anterior uveitis, lensectomy with anterior vitrectomy was performed in the right eye as previously done in the left eye. *Spiroplasma* sp. was detected in the lens and vitreous by Eubacterial 16S-rRNA sequencing and specific culture.

Postoperatively, the systemic treatment with erythromycin was extended for 3 weeks. The right eye did not show an increased inflammatory response after cataract surgery. The laboratory parameter proBNP reflecting the severity of cardiomyopathy dropped concomitantly. During long-term follow-up, the visual and general development of the child showed good progress.

On the right eye, strabismus surgery was performed in 2016. On the left eye, glaucoma surgery was needed in 2019 performing trabeculectomy with mitomycin-C (MMC).

This case has been presented as a poster on the World Congress of Paediatric Ophthalmology and Strabismus in Milan, Italy in 2012 (*7*).

Case 17

In 2013, a 3-month-old female full-term child was diagnosed with a total cataract on the right eye combined with anterior uveitis. The lens of the left eye was initially clear, but showed a progressive cataract until the age of 6 months. The child's family was living in an urban

surrounding. No abnormalities during pregnancy or delivery were documented. Lensectomy combined with anterior vitrectomy was performed in the University hospital Vienna at an age of 3 months following the same surgical protocol and postoperative treatment as case 16. *Spiroplasma* sp. was detected in the crystalline lens and the vitreous body by Eubacterial 16S-rRNA sequencing and specific culture. A systemic macrolide antibiotic (clarithromycin) was started postoperatively.

The eye was left aphakic and secondary IOL implantation was performed in 2015 on both eyes. Since 2019, the patient is under topical pressure-lowering medication on both eyes because of ocular hypertension. During the long-term follow-up, the visual and general development of the child showed good progress.

Case 18

A baby twin girl (di-amniotic, di-chorionic), born at a gestational age of 31 weeks and 3 days with a birth weight of 1580g was referred for an ophthalmological examination in the cantonal hospital of Winterthur (Switzerland) because of hazy appearing cornea at postmenstrual age of 35 weeks and 4 days. Based on the first examination, a dysgenesis of the anterior segment was presumed in the right eye, due to a white appearing membrane affecting the lens and cornea. A follow up examination two weeks later revealed anterior uveitis with a fibrinous reaction and large, irregularly shaped endothelial precipitates in the right eye and a hazy appearing endothelium in the left eye, along with posterior synechiae and immature dilated iris vessels (Figure 17). A lens opacity was not visible at this time. IOP was elevated to 23mmHg in the right eye and 19mmHg in the left eye (Tonopen).

An intensive workup including TORCHES screening did not show any cause of uveitis. Assuming a *Spiroplasma*-associated uveitis, we initiated topical anti-inflammatory (prednisolone 1%), cycloplegic (atropine 0.5%), and pressure lowering (timolol 0.25%) treatment as well as antibiotic therapy with systemic azithromycin and topical moxifloxacin for three weeks under pediatric surveillance. Due to the asymmetric inflammatory activity, the right eye was treated more intensely with steroid eye drops compared to the left eye. During the disease course, uveitis activity was under control and IOP stabilized. As the inflammation settled down and pupil

dilation improved, the lens margin was visible because of an abnormal lens shape similar to spheropakia.

The patient developed well, but a cataract developed subsequently under the topical steroid treatment. Bilateral surgical lensectomy was performed at the age of 8 months (6 months corrected age, since she was premature). A combined lens and anterior chamber fluid sample from both eyes taken at the time of lensectomy confirmed *Spiroplasma ixodetis* by 16S-rRNA PCR.

Despite topical and systemic medication, the IOP remained elevated in the left eye, requiring glaucoma surgery three months after lensectomy. A trabeculotomy was not possible due to the scarred Schlemm's canal, thus cyclophotocoagulation was performed in the left eye.

At the last follow up, 12 months after the cataract surgery, vision developed well in both eyes using aphakic contact lenses and bifocal glasses. The left eye requires amblyopia treatment (patching therapy 1-2 hours per day). Topical glaucoma treatment in the left eye is still required. No retinal complications occurred. The twin sibling did not show any signs of ocular abnormalities and is developing well.

References

1. Lorenz B, Schroeder J, Reischl U. First evidence of an endogenous *Spiroplasma* sp. infection in humans manifesting as unilateral cataract associated with anterior uveitis in a premature baby. Graefes Arch Clin Exp Ophthalmol. 2002;240:348–53. PubMed https://doi.org/10.1007/s00417-002-0453-3

2. Matet A, Le Flèche-Matéos A, Doz F, Dureau P, Cassoux N. Ocular *Spiroplasma ixodetis* in newborns, France. Emerg Infect Dis. 2020;26:340–4. PubMed https://doi.org/10.3201/eid2602.191097

3. Farassat N, Reich M, Serr A, Küchlin S, Erwemi M, Auw-Hädrich C, et al. *Spiroplasma* species as a rare cause of congenital cataract and uveitis: a case series. BMC Ophthalmol. 2021;21:434. PubMed https://doi.org/10.1186/s12886-021-02201-0

4. Martin GC, Denier M, Le Flèche-Matéos A. *Spiroplasma*-induced uveitis mimicking congenital glaucoma in a newborn. Ophthalmology. 2023;130:255. PubMed https://doi.org/10.1016/j.ophtha.2022.06.007

5. Domann E, Hong G, Imirzalioglu C, Turschner S, Kühle J, Watzel C, et al. Culture-independent identification of pathogenic bacteria and polymicrobial infections in the genitourinary tract of renal transplant recipients. J Clin Microbiol. 2003;41:5500–10. PubMed https://doi.org/10.1128/JCM.41.12.5500-5510.2003

6. Lorenz B, Andrassi-Darida M, Jäger M, Magdowski G, Imirzalioglu C. Secondary infantile cataract associated with presumed intrauterine infection. In: Poster presentation at the 36th Annual Meeting of the European Paediatric Ophthalmological Society; Bad Nauheim, Germany. 2010 Sep 20–Oct 2. Poster P17. Zürich, Switzerland: European Paediatric Ophthalmological Society; 2010.

7. Bastian FO, Sanders DE, Forbes WA, Hagius SD, Walker JV, Henk WG, et al. Spiroplasma spp. from transmissible spongiform encephalopathy brains or ticks induce spongiform encephalopathy in ruminants. J Med Microbiol. 2007;56:1235–42. PubMed https://doi.org/10.1099/jmm.0.47159-0

8. Eimer J, Fernström L, Rohlén L, Grankvist A, Loo K, Nyman E, et al. *Spiroplasma ixodetis* infections in immunocompetent and immunocompromised patients after tick exposure, Sweden. Emerg Infect Dis. 2022;28:1681–5. PubMed https://doi.org/10.3201/eid2808.212524

9. Stifter E, Moser E, Mitteregger D, et al. Pediatric cataract, anterior uveitis, and dilatative cardiomyopathy associated with *Spiroplasma* sp. Infection. Poster presentation at the 2nd World Congress of Paediatric Ophthalmology and Strabismus; Milan, Italy. 2012 Sep 7–9. London, UK: The World Society of Paediatric Ophthalmology and Strabismus; 2012.

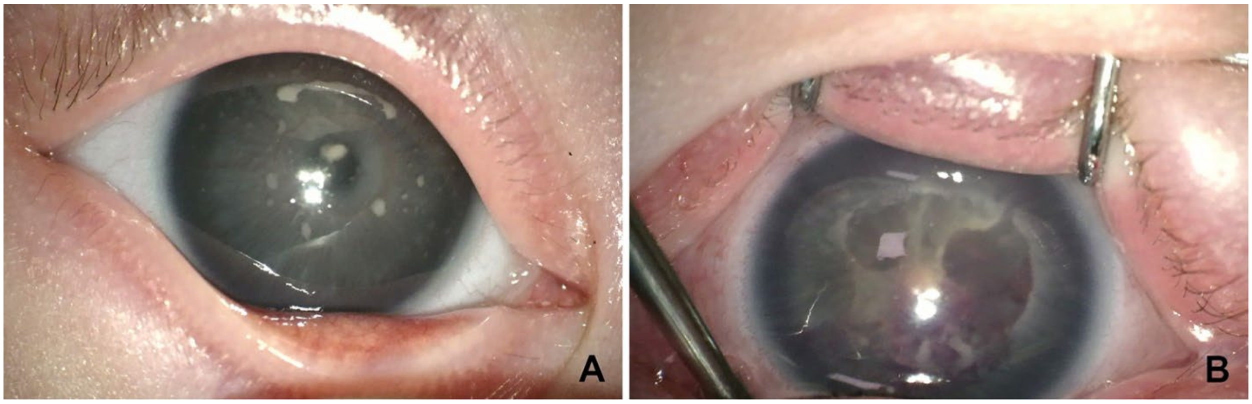

Appendix Figure 1. Eyes of patient 8 at presentation. A. Right eye at presentation with large confluent endothelial precipitates. B. Left eye at presentation with large endothelial precipitates, inflammatory membranes and blood in the anterior chamber. The corneal diameter is increased due to high intraocular pressure.

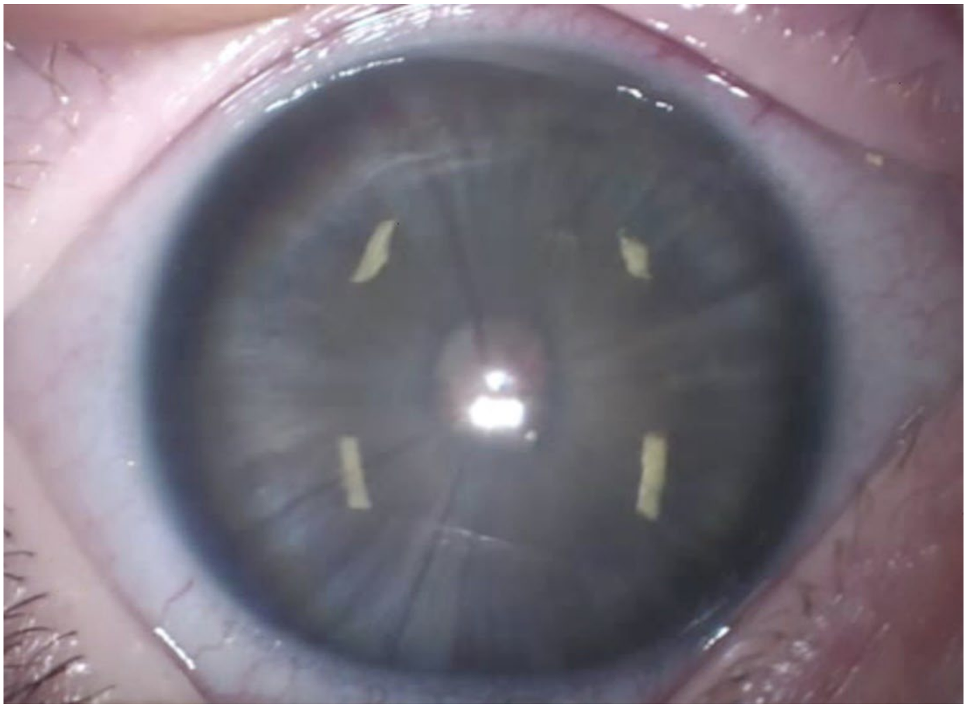

Appendix Figure 2. Patient 8, left eye, preoperative situation, showing absence of inflammation and persistence of iris vessels extending on to the lens. The four white rectangles are artefacts due to reflection of the room lights

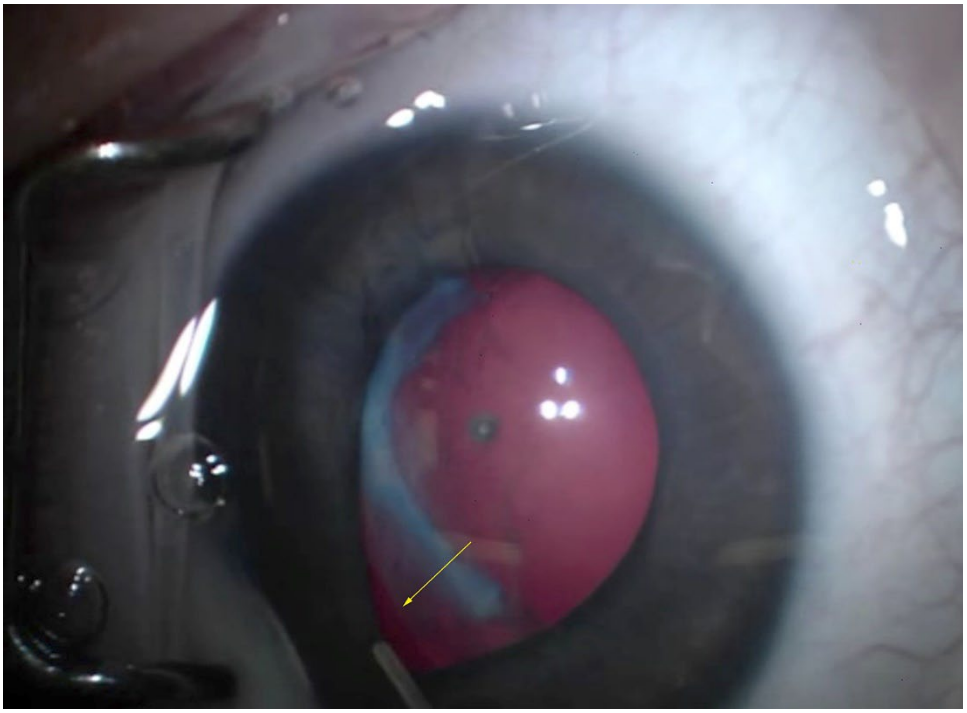

Appendix Figure 3. Patient 8, per operative situation of the right eye, seen from the temporal side. Note the absence of normal capsule formation superiorly (arrow).

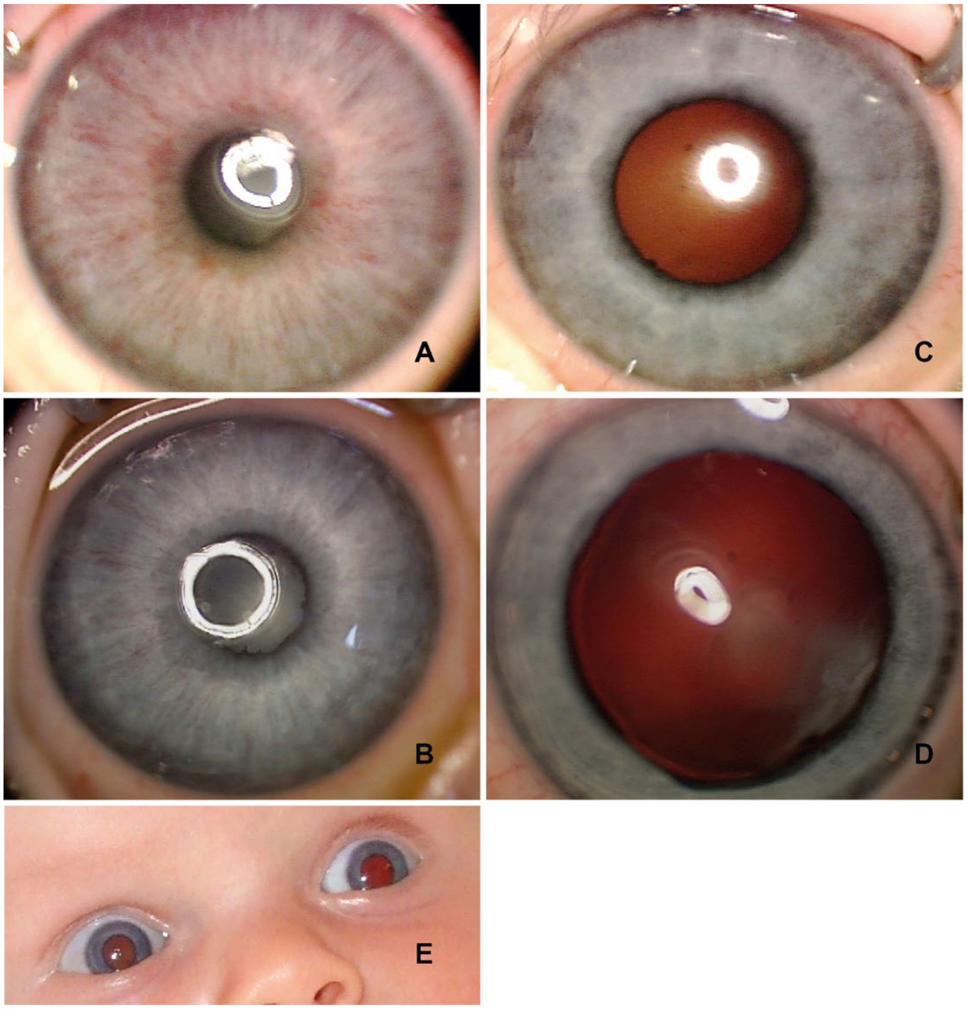

Appendix Figure 4. A. Right eye of patient 9 at presentation with severely hyperemic iris vessels. B. Left eye of patient 9 at presentation. Minimal iris hyperemia, the red reflex is slightly blunted. C. Right eye of patient 9 after anti-inflammatory treatment, the iris hyperemia has completely disappeared. D. Left eye of patient 9 after anti-inflammatory treatment, a dull red reflex and clear peripheral opacification are only visible after pupil dilation. E. Age 4 weeks, dull red reflex in the right eye.

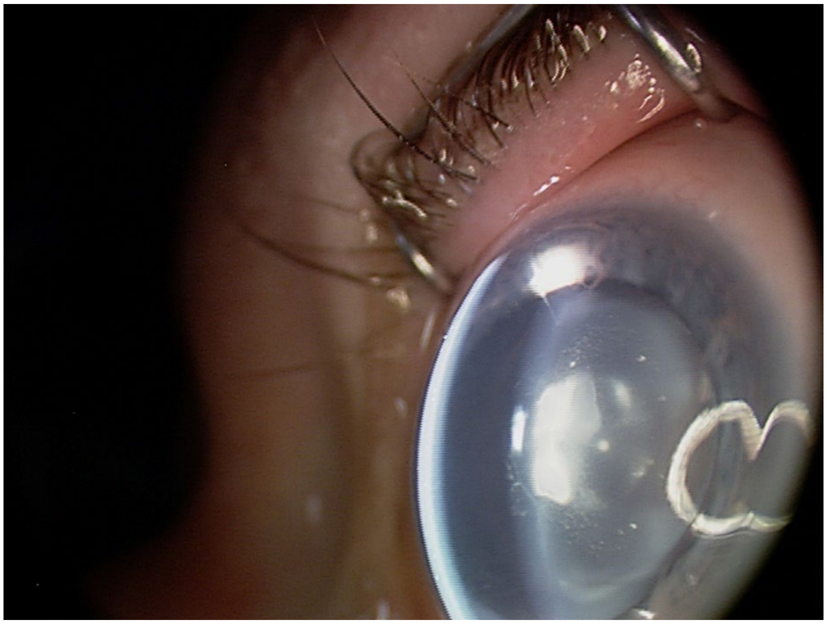

Appendix Figure 5. Left eye of patient 10 at presentation with limited iris hyperemia and evident lens opacification

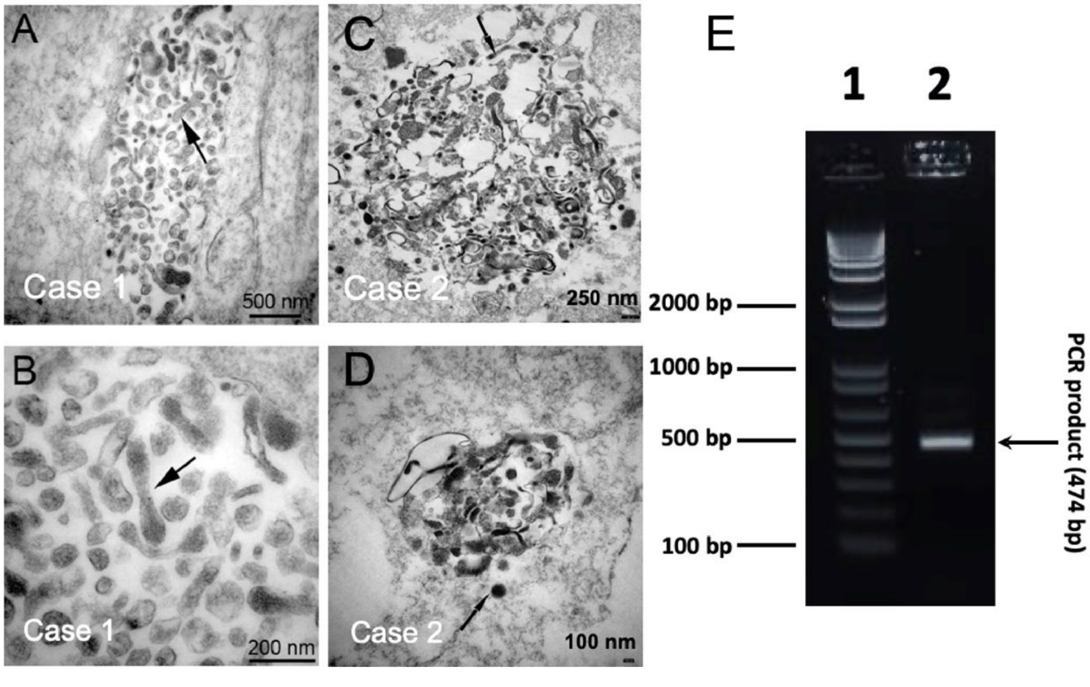

Appendix Figure 6. Transmission electron microscopy (TEM) results for patient 9 (A, B) and 10 (C, D). These show an accumulation of multiple filamentous, curved, round, and helical (arrows) profiles of *Spiroplasma* within damaged lens fiber cells at different magnifications. Note also the irregular and pleomorphic forms and the transversal barred periodicity in about one third of the *Spiroplasma* organisms. The small round profiles in image D (arrow) are probably transversely sectioned cells. E. 16S-rRNA result for case 9 indicating presence of *Spiroplasma*.

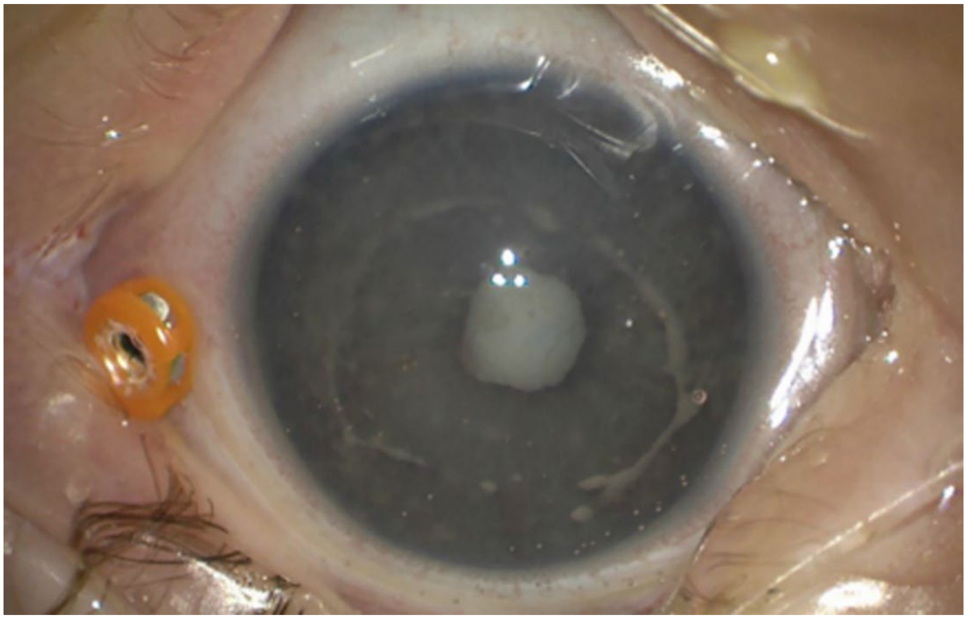

Appendix Figure 7. Peroperative image of patient 11, showing extensive corneal endothelial precipitates, posterior synechiae and white cataract.

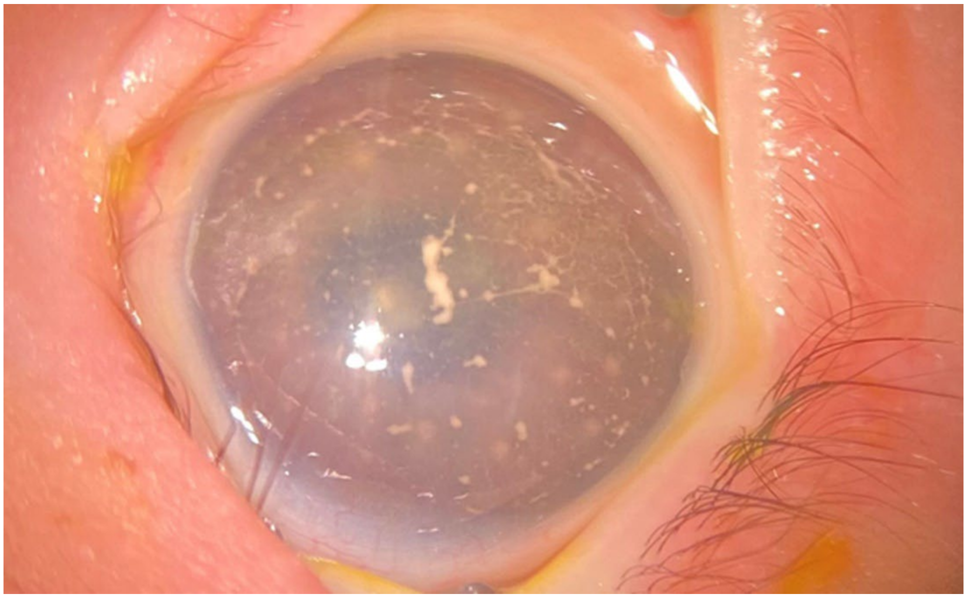

Appendix Figure 8. Examination under anesthesia of the right eye of patient 12 showed unilateral right anterior uveitis with large endothelial precipitates, iris nodules, posterior synechiae, immature dilated iris vessels and cataract.

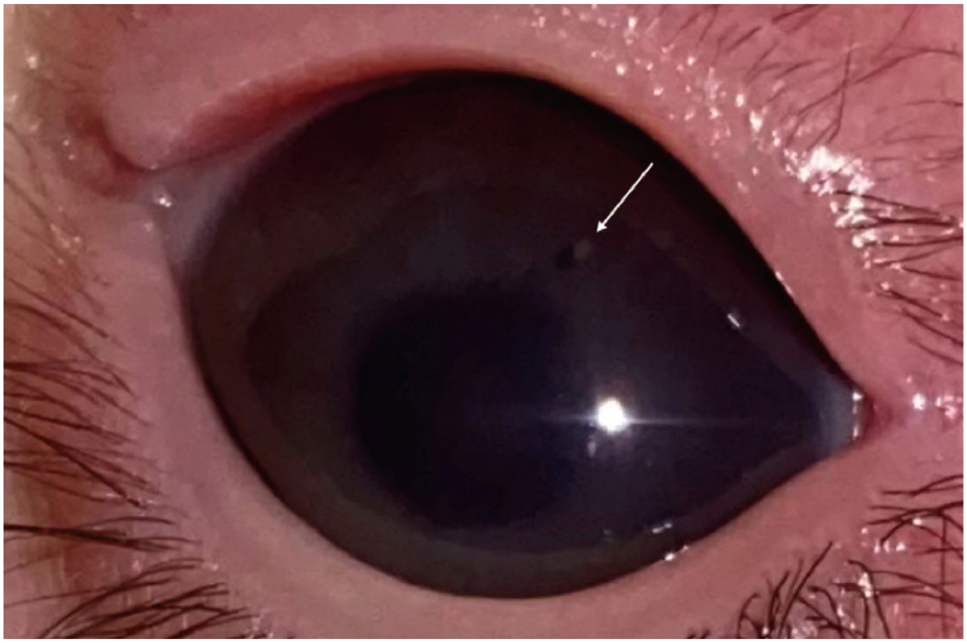

Appendix Figure 9. Ten days after the initiation of treatment, anterior chamber inflammation decreased dramatically and the fundus became partially visible. Only one endothelial precipitate remained (white arrow).

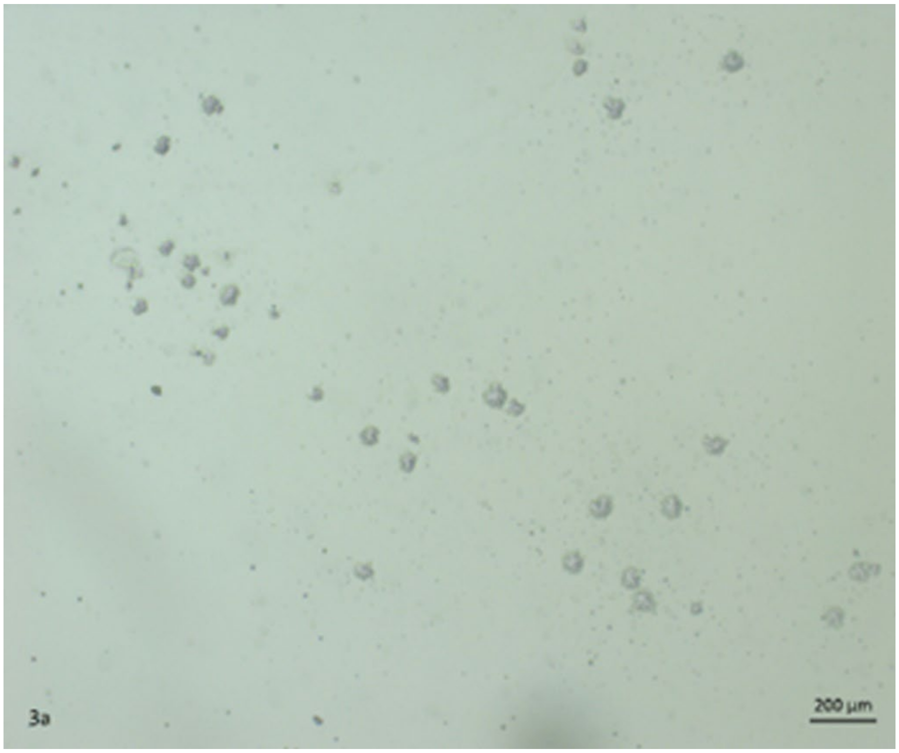

Appendix Figure 10. Growth of *S. ixodetis* isolated from fresh crystalline lens sample on Mycoplasma A7 agar medium for patient 12. Magnification, x40. Subculture (3 months).

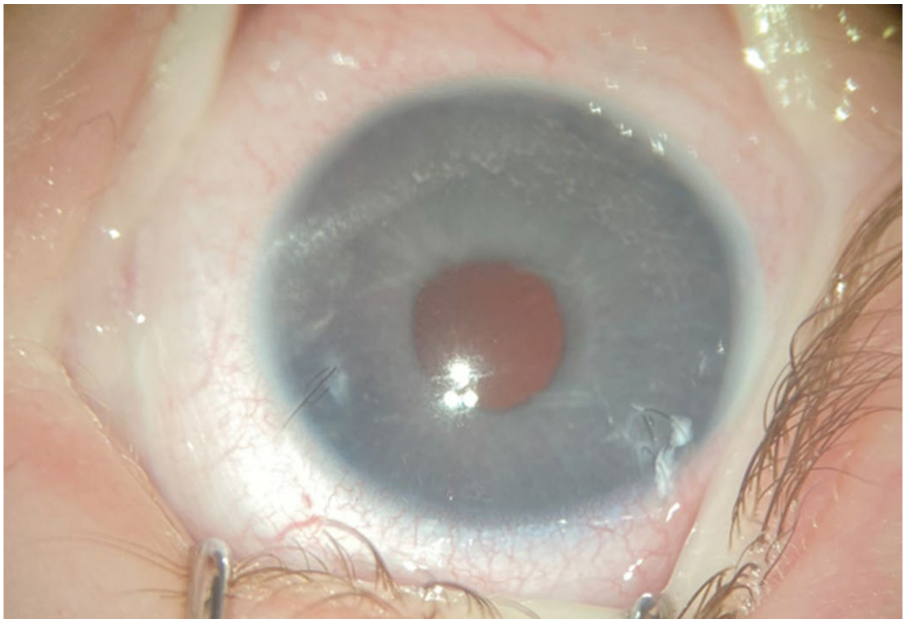

Appendix Figure 11. Inflammatory relapse in patient 13.

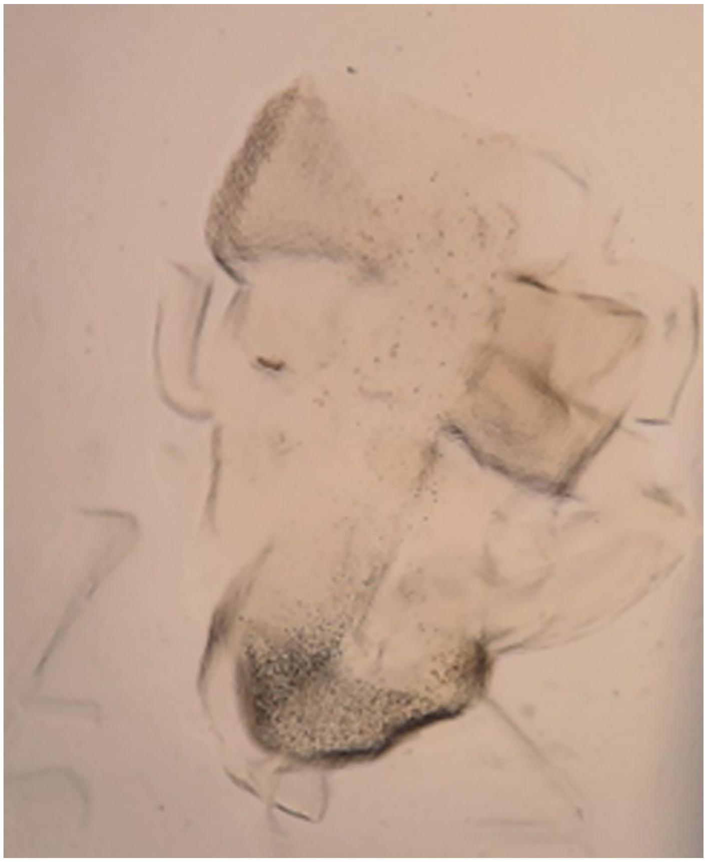

Appendix Figure 12. Growth of *S. ixodetis* isolated from fresh crystalline lens sample on Mycoplasma A7 agar medium for patient 13. Magnification, x40. Each brown and black dot represents a colony of S. ixodetis. Some fragments of crystalline lens were still visible.

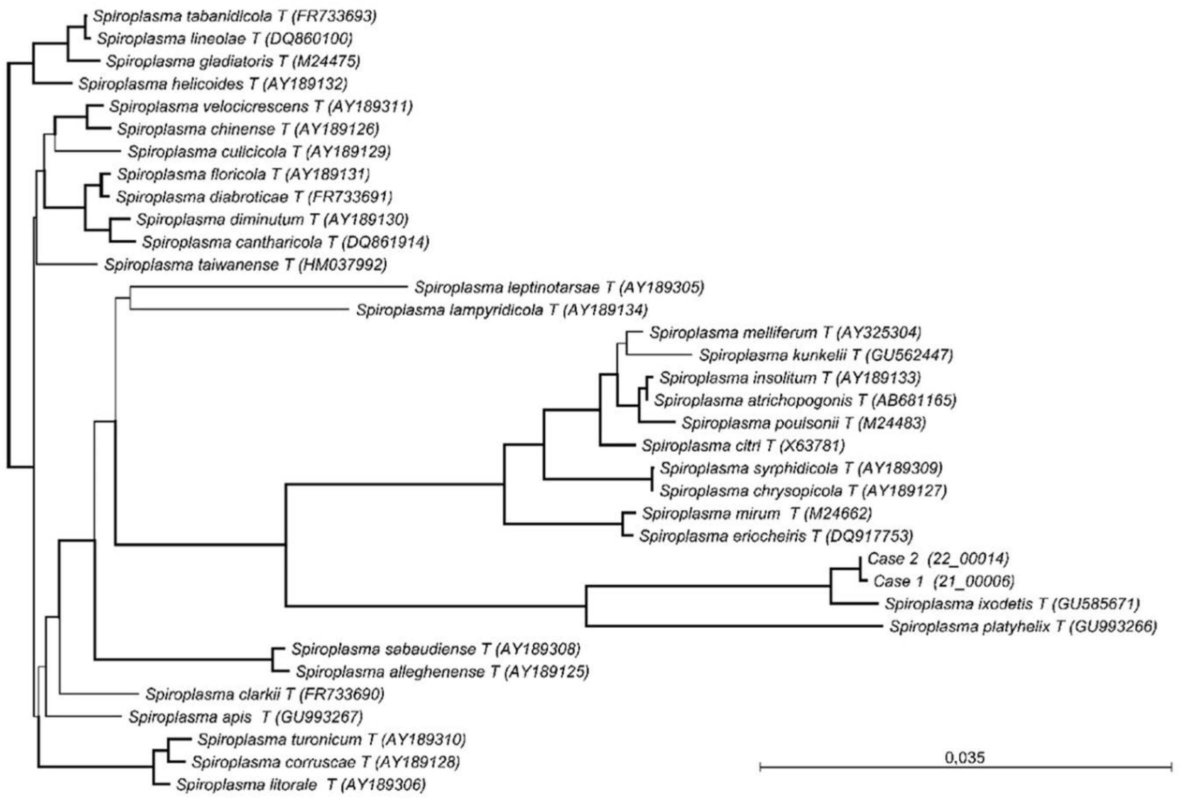

Appendix Figure 13. Neighbor-joining unrooted tree based on bacterial rrs gene sequences from the crystalline lens sample from 2 newborns (case-patient 12: “case 1”, sample 21_00006 and case-patient 13: “case2”, sample 22_00014). Thick lines indicate bootstrap values >75% (based on 1,000 replicates). Scale bar indicates the proportion of substitutions per nucleotide position.

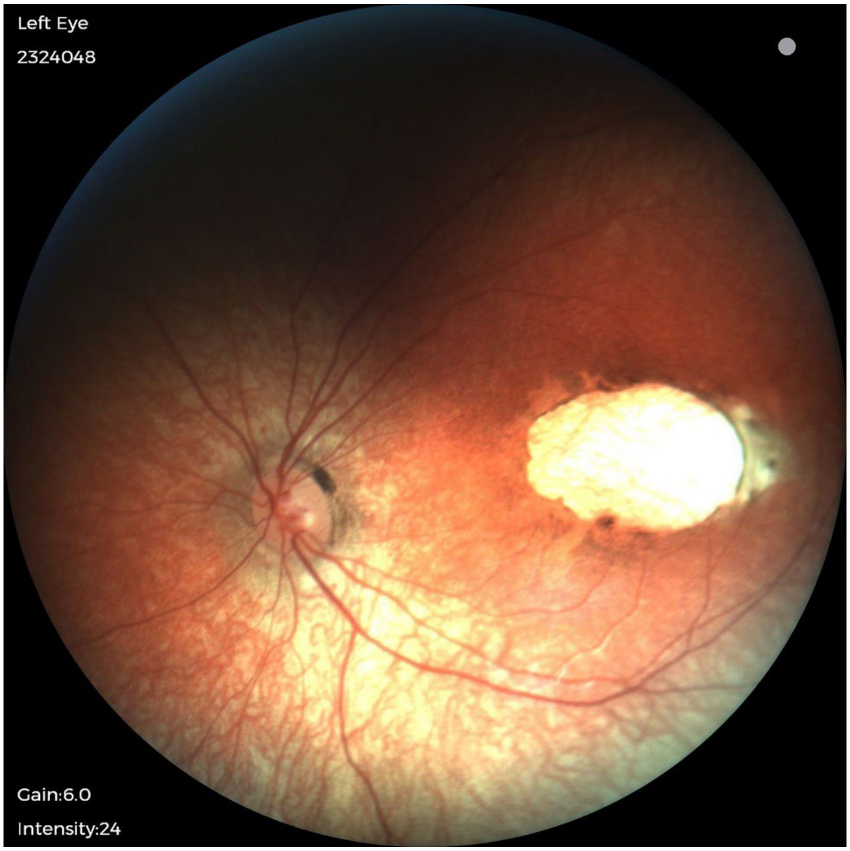

Appendix Figure 14. Fundus image of patient 14, left eye, showing a large macular scar. After removal of the cataract, a similar scar was seen in the right eye.

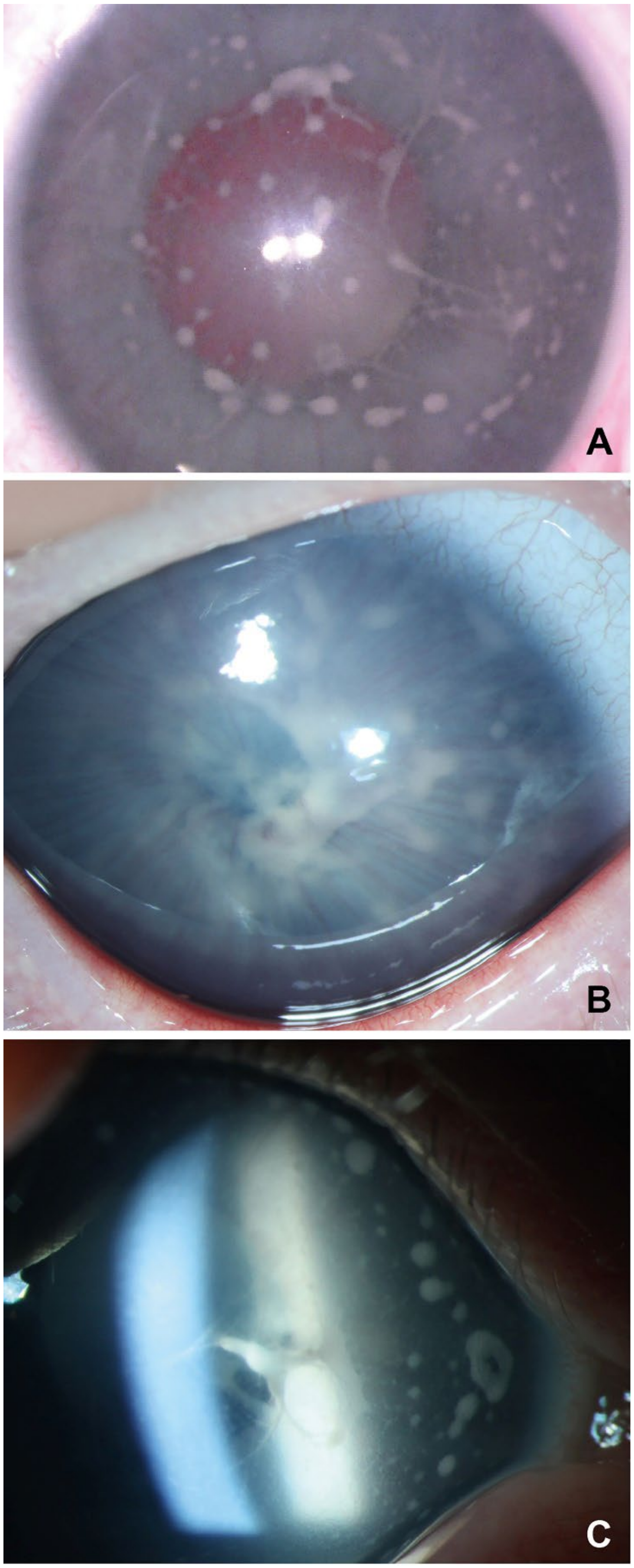

Appendix Figure 15. A, B, C. Preoperative images of case 15, showing extensive endothelial precipitates, cataract and iris vascularisation extending to the lens.

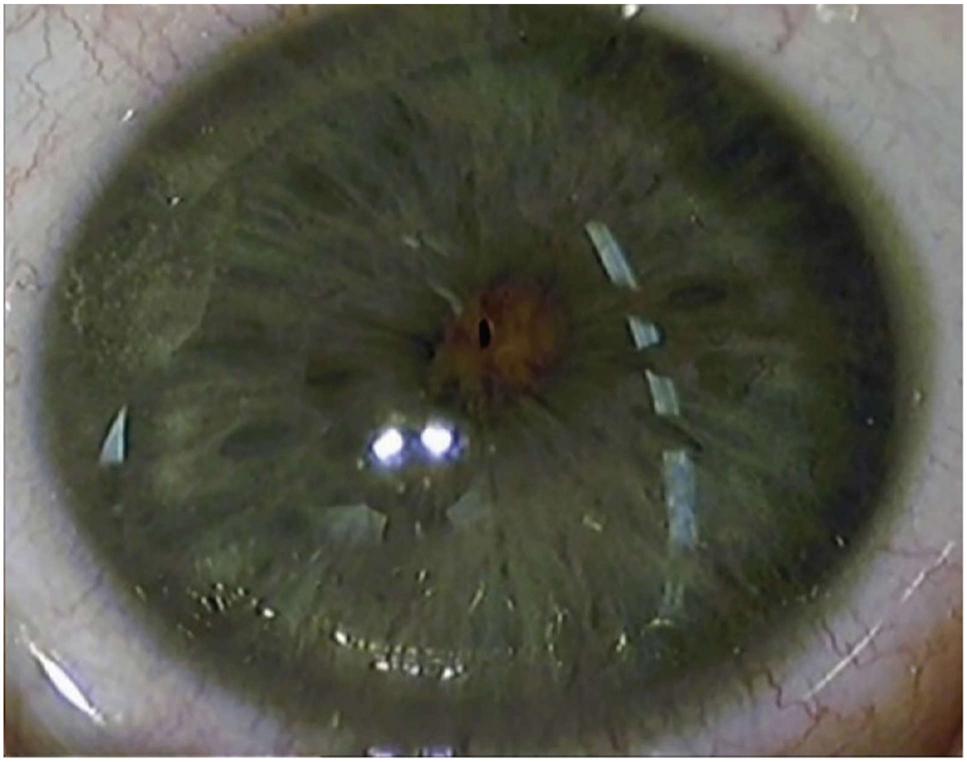

Appendix Figure 16. Preoperative image of case 16 with posterior synechiae and dilated iris vessels adherent to the anterior lens capsule

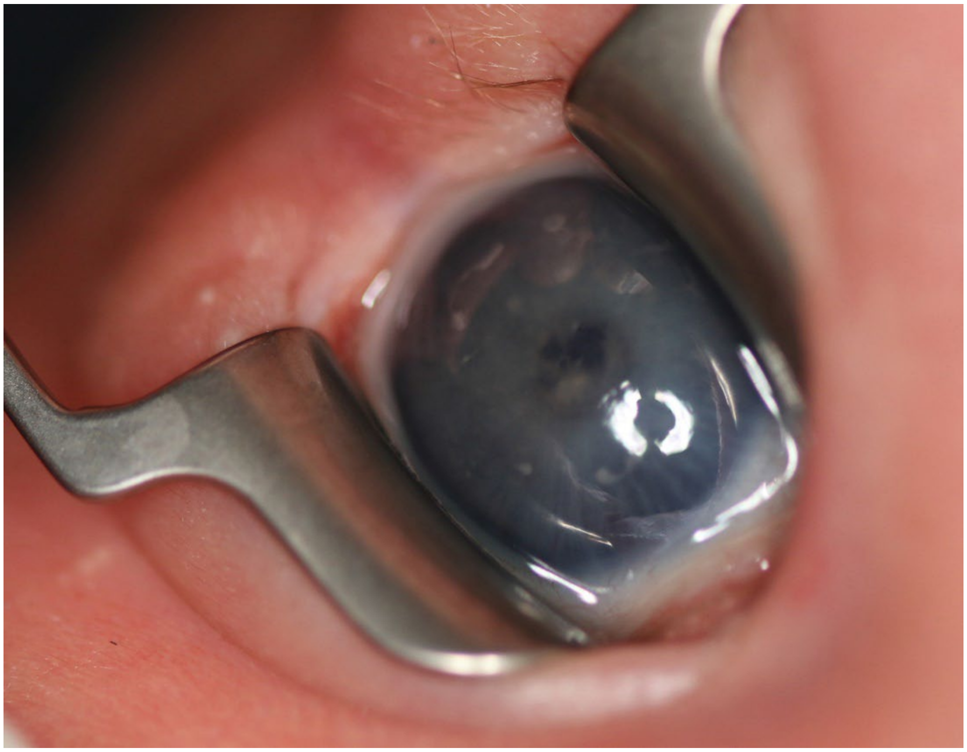

Appendix Figure 17. Right eye of patient 18 at diagnosis.
